# Storage Effects on Bioactive Phenols in Calabrian Monovarietal Extra Virgin Olive Oils Based on the EFSA Health Claim

**DOI:** 10.3390/foods12203799

**Published:** 2023-10-17

**Authors:** Marialaura Frisina, Sonia Bonacci, Manuela Oliverio, Monica Nardi, Thomas Patrizio Vatrano, Antonio Procopio

**Affiliations:** Department of Health Science, University Magna Græcia of Catanzaro, 88100 Catanzaro, Italy; m.frisina@unicz.it (M.F.); m.oliverio@unicz.it (M.O.); monica.nardi@unicz.it (M.N.); thomasvatrano@gmail.com (T.P.V.); procopio@unicz.it (A.P.)

**Keywords:** secoiridoids, extra virgin olive oil (EVOO), high-resolution mass spectrometry, method validation

## Abstract

The beneficial properties of extra virgin olive oil (EVOO) on lipids blood levels were recognized by the European Food Safety Authority (EFSA) with a health claim, specifically referring to EVOOs containing at least 5 mg of hydroxytyrosol and its secoiridoids derivatives per 20 g of oil. The main purpose of the work was to characterize the phenolic profile of two commercially available Calabrian monovarietal EVOOs (*Nocellara del Belice*, VN; *Dolce di Rossano*, VDR), and to study the effect of one-year storage on secoiridoids composition, by monthly controls. A new UHPLC-ESI-HRMS method was developed and validated, thus facilitating the EFSA claim application and allowing producers to valorize their products. Seven biologically active compounds were chosen: tyrosol, hydroxytyrosol, oleocanthal, oleacein, oleuropein aglycone, verbascoside, and oleuropein. LODs and LOQs were 0.001–0.02 mg g^−1^ and 0.002–0.08 mg g^−1^, respectively. The variation coefficients were ≤20% and the percentage of recovery was between 89–109%. During the 12-month storage period, the concentration of selected compounds ranged between 1258.78–1478.91 mg Kg^−1^ for VN, and 1408.22–2071.45 mg Kg^−1^ for VDR, with a decrease of 15% and 32% respectively. The method allows an accurate quantification of EVOO phenols thus being useful to certify the nutraceutical properties of olive oil.

## 1. Introduction

Extra Virgin Olive Oil (EVOO), the key lipid component of the Mediterranean diet, can be considered a truly functional food since the European Food Safety Authority (EFSA), just a decade ago, approved a health claim, based on solid scientific evidence [1,2], affirming that “*Olive oil polyphenols contribute to the protection of blood lipids from oxidative stress*”. The claim can be applied to EVOOs containing at least 5 mg of hydroxytyrosol and its derivatives (e.g., oleuropein complexes and tyrosol) per 20 g of olive oil [3]. EFSA’s claim makes the current classification of olive oils obsolete and insufficient, as it does not adequately describe the qualitative differences between products on the market. Indeed, the possibility of adopting an EFSA-approved health claim label, based on bioactive phenol content, would allow for the determination of the highest quality segment within the EVOO category [1,2,3,4].

The content of phenolic derivatives in freshly made EVOO depends on genotype or cultivar [5], and it is influenced by agronomic (climatic conditions, fruit ripening, irrigation practices, presence of certain pathologies, etc.) and technological factors (oil extraction process, temperature and malaxation time, added amount of water, etc.) [1,2,3,4,5].

Up to 90% of the total phenolic compounds in EVOO are represented by secoiridoids (SEC) [6] which are strongly correlated both with the bitter, fruity, green, and pungent taste of EVOO [6,7] and many biological activities, ranging from anti-inflammatory, antimicrobial, antioxidant, cytostatic effects, and antiproliferative [8,9,10,11].

**Scheme 1:**
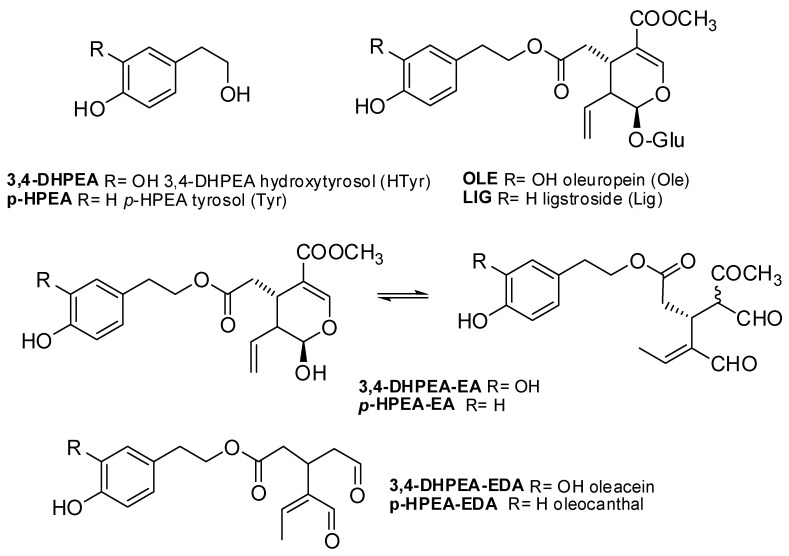
Representative structures of EVOO phenols.

Among the major SEC in olive drupes are oleuropein (OLE) and ligstroside (LIG), [esters formed by 3-hydroxytyrosol (3,4-DHPEA) or tyrosol (*p*-HPEA) and the glycosidic derivative of elenolic acid (EA)], which are accumulated in the fruit during ripening [12]. The combined action of the endogenous β-glucosidases and methylesterases, and chemical reactions occurring at different stages of olive oil production convert OLE and LIG to four major compounds both in open and closed forms: open monoaldehyde forms of oleuropein (3,4-DHPEA-EA) and ligstroside (*p*-HPEA-EA) aglycones, and their decarboxymethylated analogues 3,4-DHPEA-EDA and *p*-HPEA-EDA, usually reported as oleacein and oleocanthal, respectively (Scheme 1) [7,13,14].

However, during the EVOO storage period, the composition of phenolic compounds undergo qualitative and quantitative changes due to oxidative and hydrolytic reactions [7,15,16] that strongly depend on several variables (storage time, filtration methods, type of container, and, above all, exposure to light and high temperature) [5,7,17], and that result in a significant increase of the complexity of the EVOO phenolic content. Therefore, the lack of clarity about the bioactive phenols to be determined and of a standardized analytical method for their quantification has not yet allowed the olive oil industry to fully benefit from the EFSA claim. The officially proposed quantitative protocol expresses the total EVOO phenols, including lignans, flavonoids, and phenolic acids, as standard equivalent units (tyrosol, hydroxytyrosol, etc.) [18]. Alternatively, the determination of the EFSA-recognized phenolic contents in oils has been obtained after acid hydrolysis of all the complex tyrosol and hydroxytyrosol derivatives [4]. Such methods do not directly reflect the real content of EVOO phenols for several reasons: different responses under UV or MS detection for each phenolic compound containing tyrosol or hydroxytyrosol moieties, only partial hydrolysis of complex phenols, etc. [19]. Consequently, the quantification of these compounds is only an approximative estimation of their real concentration. Lastly, the ^1^H-NMR method proposed for the quantitative analysis of the EVOO secoiridoids [20], although it has many advantages, requires a high quantity of analytes in the sample besides a significant amount of oil sample and extraction solvents.

Therefore, a reliable quantitative analysis of phenolic secoiridoids in EVOO does not yet exist because of the limited availability of reference standards for all of them. As the EFSA claim refers to the EVOO phenols containing the hydroxytyrosol and tyrosol moieties, oleuropein, ligstroside, and their derivatives above reported (see Scheme 1), are the only phenols that should be considered. Furthermore, nuzhenide, nuzhenideoleside, and verbascoside, the other phenolic compounds of olive fruits containing 3,4-DHPEA or *p*-HPEA, have never been reported in extracted oils [21,22]. Therefore, a practical approach might be to quantify only the eight phenols containing the hydroxytyrosol and tyrosol moieties found in EVOO, which represent at least 90% of the phenols considered by the EFSA health claim and for which standards exist (see Scheme 1) [7,14,16,23]. Certifying even just the presence of these simple and complex phenols, in the quantities suitable to guarantee the beneficial effects on health declared in the EFSA claim, would assure the consumer about the quality of the purchased product.

Over the last decade, we have devoted many efforts to the development of green semi-synthetic methodologies to produce the EVOO phenols shown in Scheme 1 starting from oleuropein, which was easily extracted from the wastes of the olive oil production chain (leaves, oil mill wastewater, etc.) [8,10,11,24,25].

Therefore, we planned to employ the molecules prepared following the developed semisynthetic processes to certify the quality of an EVOO regarding the EFSA health guidelines.

The main objective of the present work was to verify the stability of secoiridoids content in two monovarietal commercial EVOOs produced by Vulcano farm, namely *Nocellara del Belice* and *Dolce di Rossano* cultivars over one year, by carrying out a monthly control (Figure S1). Both cultivars are characterized by a similar sensory profile and average total phenolic content (511–580 mg/Kg), as reported by the Italian National Database of Monovarietal Extra Virgin Olive Oils [26].

To evaluate the preservation of phenols during storage and to prove the compliance with the EFSA health claim, an ultra-high-performance liquid chromatography followed by high-resolution mass spectrometry with electrospray ionization (UHPLC-ESI-HRMS) method was developed and validated using the molecules synthesized in the house as analytical standards [8,10,25].

## 2. Materials and Methods

### 2.1. Reagents and Standards

LC-MS grade methanol (MeOH) and acetonitrile (MeCN) were purchased from VWR BDH Chemicals (Milan, Italy); n-hexane, ethanol absolute anhydrous, and formic acid 99% were purchased from Carlo Erba (Milan, Italy). Ultrapure water (18 MΩ) was obtained by a milli-Q purification system (Millipore, Bedford, MA, USA). Sodium carbonate (Na_2_CO_3_), sodium sulfate (Na_2_SO_4_), Folin-Ciocalteu reagent, and caffeic acid reference standard were purchased from Sigma-Aldrich (Milan, Italy).

Reference standards of hydroxytyrosol (3,4-DHPEA), tyrosol (*p*-HPEA), oleocanthal (*p*-HPEA-EDA), oleacein (3,4-DHPEA-EDA), oleuropein aglycone (3,4-DHPEA-EA), oleuropein, were synthesized in our laboratory as reported in previous work [10,24,25,27]. Verbascoside was purchased from Toronto Research Chemicals (Toronto, ON, Canada).

### 2.2. EVOO Samples

Twenty-four commercial samples of Calabrian monovarietal EVOOs, produced in the 2020–2021 season, were provided by Azienda Agricola Vulcano, Mirto Crosia, and Cosenza. In detail, 12 samples were obtained from the *Nocellara del Belice* cultivar (named VN) and 12 samples from *Dolce di Rossano* (named VDR). To simulate storage conditions, samples were kept away from light, at room temperature. The dark glass bottles were only opened each month just before the analysis, from February 2021 to January 2022.

### 2.3. Sample Preparation

Phenolic compounds were isolated by liquid-liquid extraction as described in our previous work [28]. A 5 g aliquot of each sample was weighed in a 50 mL conical tube and diluted with 20 mL of a MeOH/H_2_O (80/20, *v*/*v*) solution. The solution was emulsified by the Ultraturrax instrument (IKA T18 base ULTRA-TURRAX Disperser 3.561.000) for 2 min at 6000 rpm. Then, the two phases were separated by centrifugation at 4000 rpm for 15 min at room temperature. The hydroalcoholic phase was evaporated under vacuum at a maximum temperature of 30 °C in a rotary evaporator. To wash away any remaining oil, the residue was recovered with 20 mL of MeCN/hexane (1/1, *v*/*v*). After vigorous stirring, the two phases were separated, and the acetonitrile phase was brought to dryness under nitrogen flow in a vial kept in a bath at 30 °C. The dry extract was stored at −80 °C until analysis. A solution at a concentration of 100 mg L^−1^ in ethanol was obtained for subsequent LC-HRMS analysis. Each EVOO sample was extracted and analyzed in triplicate.

### 2.4. Total Phenolic Content

Total polyphenols were quantitatively measured by Folin-Ciocalteu spectrophotometric assay as reported by Iriti et al. with some modifications [29]. Briefly, 5 g of oil samples were solubilized in 5 mL of hexane and extracted in a separating funnel with 10 mL of methanol/water (80:20, *v*/*v*) three times. To remove any oil residue, 5 mL of hexane was added to the hydroalcoholic phase. The hydroalcoholic extract was then collected in a flask, dried with Na_2_SO_4_, filtered, and brought to dryness under vacuum with a rotary evaporator at a temperature of 30 °C. 5 mL of methanol was used to solubilize the dry extract, and 1 mL of the obtained solution was mixed with 5 mL of Folin-Ciocalteu reagent (diluted 1:10) in a 50 mL graduated flask. After 4 min, 4 mL of Na_2_CO_3_ solution 7.5% *w*/*v* were added, mixed vigorously, made up to volume with distilled water, and left to react for 30 min in the dark. A UV-visible spectrophotometer (UV/VIS Spectrometer Lambda 35, PerkinElmer, Waltham, MA, USA) was used to record the absorbance at 765 nm. A calibration curve of caffeic acid in a range of 25–150 μg was used (y = 0.001219x − 0.019881, R^2^ = 0.998). Total phenolic content was calculated as mg of caffeic acid equivalents per Kg (mg CAE Kg^−1^) of oil. Each sample extraction and analysis were performed in triplicate.

### 2.5. UHPLC-UV-ESI-HRMS Analysis

A reverse-phase ultra-high-performance liquid chromatography followed by high-resolution mass spectrometry, was conducted using ionization in negative mode. A Dionex Ultimate 3000 RS (Thermo Scientific—Rodano, MI, Italy), equipped with a Hypersil Gold C18 column (100 × 2.1 mm, 1.9 µm particle size, Thermo Scientific), thermostated at 24 °C, was used for UHPLC separation. The chromatographic column was equilibrated in 98% solvent A (Ultrapure water containing 0.1% of formic acid) and 2% solvent B (methanol). The solvent flow rate was maintained at 300 µL min^−1^ and the concentration of solvent B was linearly increased as follow: from 2% to 23% in 6 min, isocratic for 5 min, then increased from 23% to 50% in 7 min, and from 50% to 98% in 5 min, isocratic for 6 min, and finally returned to 2% in 6 min and isocratic for 3 min. The UV/VIS detector was set at 235, 254, 280, and 330 nm. The volume of the injected sample was 5 μL. The total run time, including column wash and equilibration, was 38 min.

A high-resolution Q-Exactive orbitrap mass spectrometer (Thermo Scientific, Rodano, MI, Italy) was employed for mass detection. Heated electrospray ionization (HESI) in negative polarity was used with the following operating conditions: 70,000 resolving power (defined as FWHM at *m*/*z* 200), IT 100 ms, and ACG target= 1 × 10^6^, scan range (100–900 *m*/*z*). In each scan, the negative exact mass [M-H]^−^ of EVOO phenols precursor ions were selected (tyrosol, 137.0608 *m*/*z*; hydroxytyrosol 153.0557 *m*/*z*; oleocanthal 303.1205 *m*/*z*; oleacein 319.1187 *m*/*z*; oleuropein aglycone 377.1248 *m*/*z*; oleuropein 539.1770 *m*/*z*, verbascoside 623.1876 *m*/*z*) in PRM (Parallel Reaction Monitoring). The operating conditions MS/MS analysis were: resolution 35.000; AGC target= 1 × 10^5^; maximum IT 200 ms; collision energy (stepped NCE) 20, 30, 40. The value of 2.0 *m*/*z* was set for the quadrupole isolation window. High-purity nitrogen was employed as both the sheath gas (30 arb units) and auxiliary gas (10 arb units). The instrument was daily calibrated before starting UHPLC-ESI-HRMS analysis using the calibration solution supplied by Thermo Fisher Scientific (Waltham, MA, USA).

The compound characterization was based on the corresponding HRMS spectra, accurate masses, characteristic fragmentations, and retention times. Xcalibur software (version 4.1) was used for instrument control, data acquisition, and data analysis.

Olive oil phenols were identified by comparison with retention times and MS data of reference standards. Reference analytical standard solutions were daily prepared in ethanol.

### 2.6. Validation of UHPLC-UV-ESI-HRMS Analysis

The UHPLC-ESI-HRMS method for identification and quantification of simple phenolic acid and their secoiridoids derivatives in olive oil samples was validated.

The sensitivity of the method was assessed by the values of limit of detection (LOD) and limit of quantification (LOQ). The LOD is defined as the lowest concentration of the analytes that can be discriminated from the blank, whereas the LOQ is the lowest concentration of each analyte detected with adequate reliability [6]. Experimental LOD and LOQ were calculated as 3× and 10× the standard deviation (SD) over ten repeated measurements of blank samples, fortified with the lowest concentration at which the compounds are detected.

A “blank” sample with a low level of EVOO phenols, was fortified with a mixture of standard compounds at three different concentration levels. Ten replicate samples were prepared at each level for a total of thirty test samples. The phenolic compounds were extracted and analyzed to estimate the repeatability and precision of the method. The behavior of the data was analyzed according to Shapiro-Wilk’s test (*p =* 0.05) to verify the normal distribution of the obtained data. In addition, Huber’s test (*p =* 0.05) was performed to check the presence of outliers, and no outliers were detected. Thus, the extended uncertainty (U_e_), the limit of repeatability (r), percentage recovery (R), and intra-day variation coefficient, expressed as CV_r_ (%), were calculated, for each compound. The inter-day variation CV_R_ (%) was evaluated by comparing the assay performed on two different days and analytical sessions. The U_e_ was obtained by multiplying the standard uncertainty by the covering factor K equal to 2 resulting from the Student’s distribution, which corresponds to a confidence interval of approximately 95%.

## 3. Results

### 3.1. Identification and Evolution of Major EVOO Phenols Profile during Storage

The first aim of the current research was the UHPLC-ESI-HRMS/MS profiling of EVOO phenols which are most related to the beneficial effects on human health, according to the EFSA claim, namely ecoiridoids and simple phenolic alcohols. Specifically, seven EVOO phenols, with well-known biological activities, were chosen among secondary metabolites: tyrosol, hydroxytyrosol, oleocanthal, oleacein, oleuropein aglycone, verbascoside, and oleuropein. Individual concentrations of the considered molecules were derived by the calibration curves of their respective standards obtained in-house, except for verbascoside, which was purchased as a commercial analytical standard.

The analysis was performed in full scan mode using parallel reaction monitoring (PRM) to select in ESI negative mode the precursor ions of investigated EVOO phenols. The preliminary tests with reference standards were performed by direct infusion, both in negative and positive mode, obtaining the best results in terms of resolution with negative ionization. For this reason, negative ionization was selected for method optimization.

**Figure 1:**
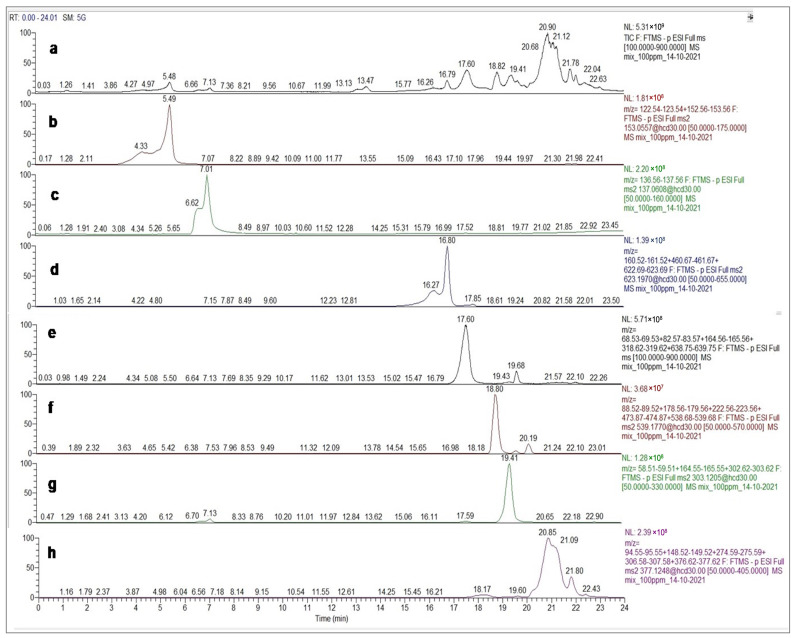
Full Scan (**a**) chromatogram obtained by UHPLC-ESI-HRMS for the [M-H]^−^ analytical mix standard and extracted ion chromatograms of hydroxytyrosol (**b**), tyrosol (**c**), verbascoside (**d**), oleacein (**e**), oleuropein (**f**), oleocanthal (**g**), and oleuropein aglycone (**h**).

Figure 1 reports the full scan and the extracted ion chromatograms of all phenols. Table 1 summarizes the daughter ions selected for quantifying and confirming each EVOO phenol.

**Figure 2:**
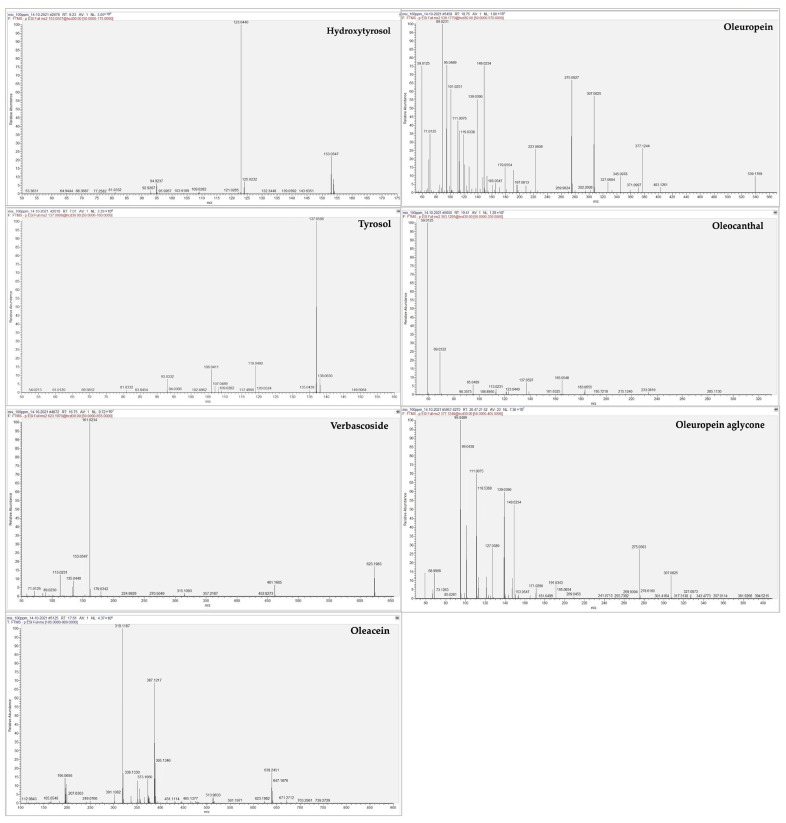
MSMS spectra obtained by UHPLC-ESI-HRMS for the [M-H]^−^ analytical standard for hydroxytyrosol, tyrosol, verbascoside, oleacein, oleuropein, oleocanthal, and oleuropein aglycone.

Figure 2 displays the mass spectra of the quantified molecules. Concerning phenolic alcohols, a peculiar product ion with *m*/*z* 123.0477, corresponding to a loss of the CH_2_OH group [30], confirmed the identification of hydroxytyrosol with *m*/*z* 153.0557. Among secoiridoids, verbascoside with molecular ion at *m*/*z* 623.1876, was characterized by the presence of distinctive fragments at *m*/*z* 461.1666 due to the loss of a hexose sugar, and at *m*/*z* 161.0234 representing the phenylpropanoid moiety due to loss of H_2_O from caffeic acid [31]. The qualifier ions detected for oleacein with *m*/*z* 195.0664 and 165.0550 correspond to C_10_H_11_O_4_ and C_9_H_9_O_3_ respectively, as also reported by Luque-Muñoz et al. [32]. A similar fragment at *m*/*z* 165.0548 corresponding at C_9_H_9_O_3_, was observed for oleocanthal [33]. It is important to highlight that oleuropein aglycone, having a *m*/*z* 377.1248, was detected not as a single peak but as a remarkable number of peaks due to different isomer forms, as other authors have already reported [14]. It was characterized by a fragmentation pattern of *m*/*z* 307.0825 due to the elimination of a C_4_H_6_O neutral fragment, *m*/*z* 275.0927 for a further loss of an –OCH_3_ moiety, and *m*/*z* 149.0234 due to a further loss of a phenyl moiety and closure of the ends of the heterocyclic ring, as previously reported by Kanakis et al. [34]. After cleavage of hexose sugar, oleuropein fragmentation shares some common daughter ions with its aglycone.

**Figure 3:**
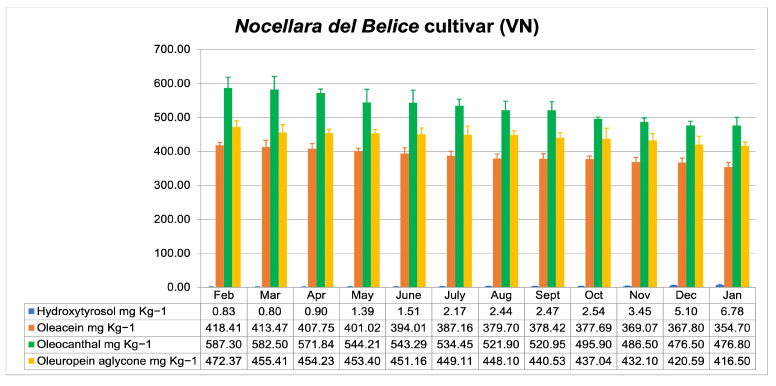
Evolution of hydroxytyrosol, oleacein, oleocanthal, and oleuropein aglycone concentrations (mg Kg^−1^) during twelve months of storage in *Nocellara del Belice* monovarietal EVOO.

**Figure 4:**
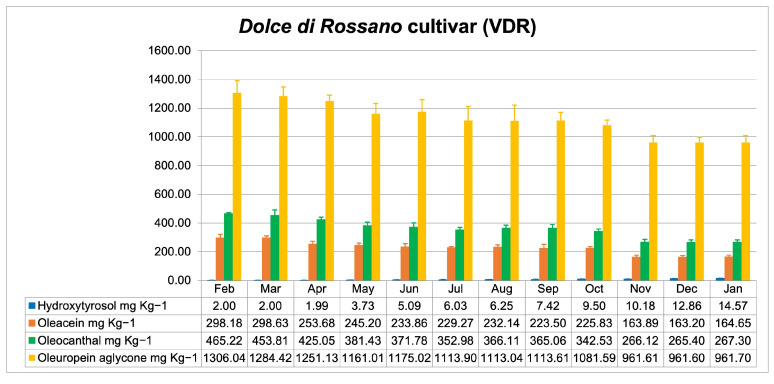
Evolution of hydroxytyrosol, oleacein, oleocanthal, and oleuropein aglycone concentrations (mg Kg^−1^) during twelve months of storage in *Dolce di Rossano* monovarietal EVOO.

Figure 3 and Figure 4 show the quantification of EVOO phenols in both VN and VDR monovarietal olive oil samples. All data are expressed as mg Kg^−1^ of oil using the following equation:

$$C\left(\mathrm{mg}/\mathrm{Kg}\right)=\frac{\frac{c\left(\mathrm{mg}/L\right)\;\times \;A\;\left(\mathrm{mg}\right)}{100\;\mathrm{mg}/L}}{B\;\left(\mathrm{Kg}\right)}$$

For each sample, the concentration (*C*) was calculated considering the weight of the hydroalcoholic extract (***A*** expressed in mg), the concentration of extract solution obtained for analysis equal to 100 mg L^−1^, and the initial weight of the oil sample (***B*** expressed in Kg). The individual concentrations of each compound (*c*), reported in mg L^−1^, were calculated by the external calibration curves drawn from their respective analytical standard (see Figure S3). Among the considered EVOO phenols, oleuropein, and verbascoside were not detected, as already reported by other authors [21,22], while tyrosol concentration was found to be below the detection limit in all samples. In both sets of samples, the other secoiridoid derivatives mostly contributed to the total EVOO phenols content, while the concentration of hydroxytyrosol did not exceed 15 mg Kg^−1^.

The total quantified EVOO phenols in oil samples, provisionally calculated as the sum of the individual concentrations, ranged from 1478.91 mg Kg^−1^ to 1258.78 mg Kg^−1^ for *Nocellare del Belice*, and from 2071.45 mg Kg^−1^ to 1408.22 mg Kg^−1^ for *Dolce di Rossano*. Therefore, a decrease of 15% and 32% was assessed in VN and VDR, respectively, after storage for 12 months.

**Figure 5:**
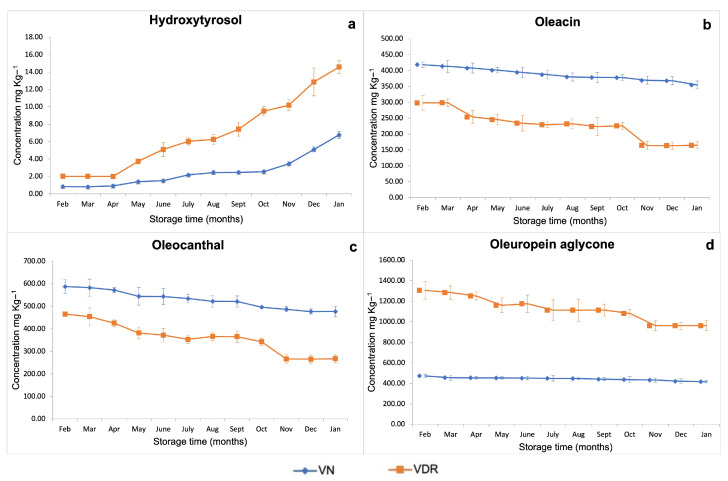
Time course of individual EVOO phenols during storage time (months) in *Nocellara del Belice* (VN) and *Dolce di Rossano* (VDR) monovarietal EVOOs: (**a**) hydroxytyrosol, (**b**) oleacein, (**c**) oleocanthal, and (**d**) oleuropein aglycone.

As regards the individual compounds (Figure 5), simple phenolic alcohol hydroxytyrosol increased its concentration throughout storage in parallel with the decrease of secoiridoid derivatives. Comparing the two cultivars, VN samples appear to be more stable compared to VDR, considering that the concentration of hydroxytyrosol underwent a smaller increase over time. In addition, VN samples showed a higher initial concentration of oleacein and oleocanthal, which were more stable over time than in VDR samples. On the contrary, VDR samples showed a lower initial concentration of oleacein and oleocanthal, and their greater reduction was observed. In detail, a decrease of 15% and 19% was assessed in VN for oleacein and oleocanthal respectively, whereas a 45% and 43% reduction was observed in VDR samples. In contrast to VN samples, the secoiridoid that mostly contributed to the total EVOO phenol concentration in VDR was oleuropein aglycone.

Finally, total phenol content in monovarietal EVOO samples was evaluated by a spectrophotometric assay (Folin-Ciocalteu) showing an average amount of 509.173 mg kg^−1^ and 527.843 mg kg^−1^ in VN and VDR samples respectively (Table S1), with a minimal variability during the storage period.

### 3.2. Analytical Method Validation

The method was validated in terms of linearity, LODs, LOQs, precision, and accuracy. The results of the external analytical standards showed good linearity between the peak area obtained by UHPLC-ESI-HRMS and the concentration of analyte (see Figure S3), showing correlation coefficients (R^2^) between 0.9902 and 0.9987 (Table S3).

To evaluate LODs, LOQs, and precision, three different concentration levels for each compound were first considered (see Table S4). The normal distribution of data was confirmed by Shapiro-Wilk’s test, whereas Huber’s test verified the absence of outliers.

As shown in Table 2, LOD values were between 0.001 and 0.02 mg g^−1^, and LOQs ranged from 0.002 to 0.08 mg g^−1^.

As already reported by Bellumori et al. [4], precision and accuracy for each EVOO phenol were calculated by variation coefficient (CV_r_ and CV_R_%) and percentage of recovery (R%), respectively. The variation coefficients, reported in Table 2, are the highest values considered over the three concentration levels. The CV_r_ and CV_R_ ranged from 5.8% to 9.7%, and from 6.6% to 10.9%, respectively, thus ≤20% in all samples. The R values were between 89% and 109% (Table 2), therefore within the 80–120% range. These results are in accordance with the International Organization for Standardization [35] and in line with the results previously shown in the literature [4,6].

## 4. Discussion

The focus of the present research was to propose a new method to assess the stability of EVOO phenols content in two Calabrian monovarietal oils, during a storage period of 12 months, by performing a monthly control. This could help producers in assessing oil shelf life and compliance with the EFSA health claim, considering that during storage, EVOO phenols experienced qualitative and quantitative changes. *Nocellara del Belice* and *Dolce di Rossano* have been selected among the most widespread cultivars in Calabria, for their well-balanced bitter and sweet taste attributes, and for the high content of phenols that is almost twice the amount required by EFSA.

Our attention has been focused on the identification and quantification of seven bioactive EVOO phenols, namely tyrosol, hydroxytyrosol, oleocanthal, oleacein, oleuropein aglycone, verbascoside and oleuropein, using analytical standards synthesized in house [10,24,25,27]. One of the current issues in developing reproducible analysis for the phenolic fraction of EVOO is the lack of pure standards, therefore green semisynthetic methods have been developed in our lab to obtain the compounds of interest, which were obtained in high purity to be used as analytical standards. It is worth noting that the reason why ligstroside and its aglycone form were still not included in the method is that our research group is still working on optimizing their synthesis and/or extraction.

The selected compounds were identified by high-resolution mass spectrometry, monitoring the specific precursor ions and the corresponding characteristic fragments, as already reported (see Figure 1, Figure 2 and Figure S2) [14,30,31,32,33,34].

As expected regarding phenols quantification, and according to the literature above mentioned, oleuropein and verbascoside were not detected, due to their high hydrophilicity and degradability [21,22,28]. Tyrosol concentration was found to be below the detection limit in all samples. Similar trends were previously reported by Castillo-Luna et al. [7]. Therefore, these compounds were considered in the method validation but not included in this study.

Although VDR had higher initial and final concentrations of total EVOO phenols, their decrease over time was consistently higher than that observed in VN (15% vs. 32% at the end of the monitoring time, respectively). However, the decrease in EVOO phenols in both cultivars is lower than that generally reported in the literature, which is approximately 40% and 70% after 12 or 24 months of storage [7,36,37].

Regarding the increase in hydroxytyrosol concentration over time, this trend could be attributed to the degradation of hydroxytyrosol-conjugated compounds during the storage, naturally occurring as a result of hydrolysis and oxidation processes. Indeed, secoiridoids are compounds in which simple phenolic alcohols are linked with an ester bound to the aldehydic forms of decarboxymethyl elenolic acid, glycosylated or not, and the lysis of this conjugated compound results in an increase of the simple phenols [17]. However, the decrease in the concentration of secoiridoid derivatives and the simultaneous increase of hydroxytyrosol is not proportional, hence, as already reported, no direct conversion was observed [7].

VN samples exhibited a higher initial concentration of oleacein and oleocanthal, and a greater stability of total phenol content over time, as compared to VDR oil, in contrast to the results of Castillo-Luna et al. [7], who reported that EVOOs with high levels of oleacein and oleocanthal are subjected to large degradation processes. This evidence was confirmed by the more significant increase of hydroxytyrosol concentration observed in VDR, suggesting enhanced degradation of its derivatives. In particular, in VDR samples the hydroxytyrosol-conjugated secoiridoid that mostly contributed to the total amount of phenols was oleuropein aglycone, and its concentration underwent a larger reduction over time, as compared to VN, thus contributing to the hydroxytyrosol increase.

Our results are in line with the study of Gomez et al., in which a higher reduction of polyphenols occurred in EVOOs with a greater initial secoiridoids content [38], contrary to the trend reported by Esposto et al. [37,39].

Finally, to promote the application of the EFSA health claim on EVOO phenols and better characterize monovarietal EVOOs, more specific analytical procedures should be applied to investigate the secoiridoids composition of extra-virgin olive oils. In this regard, the method proposed showed good linearity, LODs, and LOQs values, as above described. Furthermore, data obtained for precision and accuracy, in terms of variation coefficient and percentage of recovery, are in line with the International Organization for Standardization and with the literature [4,6,35].

## 5. Conclusions

The present work proposed a reliable analytical method to identify and quantify the EVOO phenols in the oil during storage conditions. In particular, two local monovarietal EVOO samples, namely *Nocellara del Belice* and *Dolce di Rossano*, were analyzed to assess their initial content in EVOO phenols and monitor the evolution of their concentration during a 12-month storage period.

Dosing polyphenols is a useful tool for producers to inform and ensure consumers about the high nutritional quality of EVOO. As reported by the EFSA health claim, the beneficial effect of EVOOs is achieved by a daily intake of 20 g of olive oil containing at least 5 mg of hydroxytyrosol and its derivatives. Our data demonstrated that both olive oils analyzed are in line with the EFSA claim during the monitoring year (see Table S2).

Therefore, we can assume that the producer could bear the claim on the label and this certification is valid for one year. Moreover, our data confirm the increase of hydroxytyrosol concentration during the storage period, therefore this phenolic alcohol represents a discriminating factor in recognizing recently produced EVOOs from aged ones, to identify any possible food fraud.

In the absence of an official analytical method, the proposed approach allows an accurate identification and quantification of EVOO phenols to certify the potential nutraceutical properties of olive oil. To finalize the method, future goals will be to include in the analysis the other EVOO secoiridoids, currently not quantified because of the lack of analytical standards.

## Figures and Tables

**Table 1:** EVOO phenols were investigated and quantified in EVOO samples.

Identification	Molecular Formula	[M-H]^−^ (*m*/*z*)	Main Detected Fragments (*m*/*z*)
Hydroxytyrosol	C_8_H_9_O_3_	153.0557	123.0447
Tyrosol	C_8_H_9_O_2_	137.0608	-
Verbascoside	C_29_H_35_O_15_	623.1876	461.1666 161.0234
Oleacein	C_17_H_19_O_6_	319.1187	165.0550 195.0664
Oleuropein	C_25_H_31_O_13_	539.1770	377.1244 307.0825 275.0927
Oleocanthal	C_17_H_19_O_5_	303.1205	165.0548 59.0125
Oleuropein aglycone	C_19_H_21_O_8_	377.1248	307.0825 275.0927 149.0234

**Table 2:** Statistical and analytical parameters: limit of detection (LOD); limit of quantification (LOQ); variation coefficients (CVr and CV_R_); limit of repeatability (r); percentage recovery (R), extended uncertainty (Ue).

	LOD	LOQ	CV_r_ (%)	CV_R_ (%)	r (%)	R (%)	U_e_ (%)
**Hydroxytyrosol**	0.001	0.002	6.6	7.3	21	93–109	33
**Tyrosol**	0.02	0.08	6.8	7.7	21	96–97	36
**Verbascoside**	0.001	0.002	9.3	9.9	29	97–104	29
**Oleacein**	0.01	0.03	7.5	8.0	24	94–108	33
**Oleuropein**	0.001	0.002	9.7	10.9	32	89–104	38
**Oleocanthal**	0.01	0.03	5.8	6.6	19	93–97	39
**Oleuropein aglycone**	0.02	0.08	6.8	7.2	22	95–98	36

## Data Availability

The data presented in this work are available upon request from the corresponding authors.

## Supplementary Materials

The following supporting information can be downloaded at: https://www.mdpi.com/article/10.3390/foods12203799/s1, Figure S1. Images from https://www.olimonovarietali.it/en/region-detail/?regione=CALABRIA (accessed on 13 September 2023); Figure S2. Full Scan (a) chromatogram obtained by UHPLC-ESI-HRMS for the VN sample and extracted ion chromatograms of detected EVOO phenols; Table S1. Evolution of total phenol content (mg kg^−1^) in VN and VDR monovarietal EVOO samples; Table S2. Evolution of total considered biophenols during twelve months of storage expressed as mg of EVOO phenols per 20 g in VN and VDR monovarietal EVOO samples (mg/20 g); Figure S3. Correlation of the peak area obtained by UHPLC-ESI-HRMS and analytical standard concentrations; Table S3. Linearity validation results of the analytical method by UHPL-ESI-HRMS; Table S4. Statistical and analytical parameters: variation coefficient (CV_r_ and CV_R_); limit of repeatability (r); percentage recovery (R) and extended uncertainty (U_e_) for each considered concentration (mg g^−1^).

## References

[1] Covas M.I., de la Torre K., Farré-Albaladejo M., Kaikkonen J., Fitó M., López-Sabater C., Pujadas-Bastardes M.A., Joglar J., Weinbrenner T., Lamuela-Raventós R.M. (2006). Postprandial LDL phenolic content and LDL oxidation are modulated by olive oil phenolic compounds in humans. Free Radic. Biol. Med..

[2] de la Torre-Carbot K., Chávez-Servín J.L., Jaúregui O., Castellote A.I., Lamuela-Raventós R.M., Nurmi T., Poulsen H.E., Gaddi A.V., Kaikkonen J., Zunft H.F. (2010). Elevated circulating LDL phenol levels in men who consumed virgin rather than refined olive oil are associated with less oxidation of plasma LDL. J. Nutr..

[3] EFSA Panel on Dietetic Products, Nutrition and Allergies (NDA) (2011). Scientific Opinion on the substantiation of health claims related to polyphenols in olive and protection of LDL particles from oxidative damage (ID 1333, 1638, 1639, 1696, 2865) pursuant to Article 13(1) of Regulation (EC) No. 1924/2006. EFSA J..

[4] Bellumori M., Cecchi L., Innocenti M., Clodoveo M.L., Corbo F., Mulinacci N. (2019). The EFSA Health Claim on Olive Oil Polyphenols: Acid Hydrolysis Validation and Total Hydroxytyrosol and Tyrosol Determination in Italian Virgin Olive Oils. Molecules.

[5] Kotsiou K., Tasioula-Margari M. (2016). Monitoring the phenolic compounds of Greek extra-virgin olive oils during storage. Food Chem..

[6] Lozano-Castellón J., López-Yerena A., Olmo-Cunillera A., Jáuregui O., Pérez M., Lamuela-Raventós R.M., Vallverdú-Queralt A. (2021). Total Analysis of the Major Secoiridoids in Extra Virgin Olive Oil: Validation of an UHPLC-ESI-MS/MS Method. Antioxidants.

[7] Castillo-Luna A., Criado-Navarro I., Ledesma-Escobar C.A., López-Bascón M.A., Priego-Capote F. (2021). The decrease in the health benefits of extra virgin olive oil during storage is conditioned by the initial phenolic profile. Food Chem..

[8] Sindona G., Caruso A., Cozza A., Fiorentini S., Lorusso B., Marini E., Nardi M., Procopio A., Zicari S. (2012). Anti-Inflammatory Effect of 3,4-DHPEA-EDA [2-(3,4-Hydroxyphenyl) ethyl (3S, 4E)-4-Formyl-3-(2-Oxoethyl)Hex-4-Enoate] on Primary Human Vascular Endothelial Cells. Curr. Med. Chem..

[9] Juli G., Oliverio M., Bellizzi D., Gallo Cantafio M.E., Grillone K., Passarino G., Colica C., Nardi M., Rossi M., Procopio A. (2019). Anti-tumor activity and epigenetic impact of the polyphenololeacein in multiple myeloma. Cancers.

[10] Mancuso S., Costanzo P., Bonacci S., Nardi M., Oliverio M., Procopio A. (2021). Green SemisyntheticCascade to Ligstroside, LigstrosideAglycone, and Oleocanthal. ACS Sustain. Chem. Eng..

[11] Oliverio M., Nardi M., Di Gioia M.L., Costanzo P., Bonacci S., Mancuso S., Procopio A. (2021). Semi-synthesis as a tool for broadening the health applications of bioactive olive secoiridoids: A critical review. Nat. Prod. Rep..

[12] Jimenez B., Sanchez-Ortiz A., Lorenzo M.L., Rivas A. (2013). Influence of fruit ripening on agronomic parameters, quality indices, sensory attributes and phenolic compounds of Picudo olive oils. Food Res. Int..

[13] Diamantakos P., Velkou A., Killday K.B., Gimisi T., Melliou E., Magiatis P. (2015). Oleokoronal and oleomissional: New major phenolic ingredients of extra virgin olive oil. OLIVAE.

[14] Abbattista R., Losito I., Castellaneta A., De Ceglie C., Calvano C.D., Cataldi T.R.I. (2020). Insight into the Storage-Related Oxidative/Hydrolytic Degradation of Olive Oil Secoiridoids by Liquid Chromatography and High- Resolution Fourier Transform Mass Spectrometry. J. Agric. Food Chem..

[15] Krichene D., Salvador M.D., Fregapane G. (2015). Stability of Virgin Olive Oil Phenolic Compounds during Long-Term Storage (18 Months) at Temperatures of 5–50 degrees C. J. Agric. Food Chem..

[16] Esposto S., Taticchi A., Servili M., Urbani S., Sordini B., Veneziani G., Daidone L., Selvaggini R. (2021). Overall quality evolution of extra virgin olive oil exposed to light for 10 months in different containers. Food Chem..

[17] Lozano-Sánchez J., Bendini A., Quirantes-Piné R., Cerretani L., Segura-Carretero A., Fernández-Gutiérrez A. (2013). Monitoring the bioactive compounds status of extra-virgin olive oil and storage by-products over the shelf life. Food Control.

[18] (2009). Determination of Biophenols in Olive Oils by HPLC.

[19] Celano R., Piccinelli A.L., Pugliese A., Carabetta S., Di Sanzo R., Rastrelli L., Russo M. (2018). Insights into the Analysis of Phenolic Secoiridoids in Extra Virgin Olive Oil. J. Agric. Food Chem..

[20] Karkoula E., Skantzari A., Melliou E., Magiatis P. (2014). Quantitative measurement of major secoiridoid derivatives in olive oil using qNMR. Proof of the artificial formation of aldehydic oleuropein and ligstroside aglycon isomers. J. Agric. Food Chem..

[21] Silva S., Gomes L., Leitão F., Bronze M., Coelho A.V., Boas L.V. (2010). Secoiridoids in olive seed: Characterization of nüzhenide and 11-methyl oleosides by liquid chromatography with diode array and mass spectrometry. Grasas Y Aceites.

[22] Trapani S., Breschi C., Cecchi L., Guerrini L., Mulinacci N., Parenti A., Canuti V., Picchi M., Caruso G., Gucci R. (2017). Indirect indices of oxidative damage to phenolic compounds for the implementation of olive paste malaxation optimization charts. J. Food Eng..

[23] Miho H., Moral J., Lopez-Gonzalez M.A., Diez C.M., Priego-Capote F. (2020). The phenolic profile of virgin olive oil is influenced by malaxation conditions and determines the oxidative stability. Food Chem..

[24] Procopio A., Alcaro S., Nardi M., Oliverio M., Ortuso F., Sacchetta P., Pieragostino D., Sindona G. (2009). Synthesis, biological evaluation, and molecular modeling of oleuropein and its semisynthetic derivates as cyclooxygenase inhibitors. J. Agric. Food Chem..

[25] Costanzo P., Bonacci S., Cariati L., Nardi M., Oliverio M., Procopio A. (2018). Simple and Efficient Sustainable Semi-synthesis of oleacein [2-(3,4-hydroxyphenyl) ethyl (3S,4E)-4-formyl-3-(2-oxoethyl)hex-4-enoate] as potential additive for edible oils. Food Chem..

[26] https://www.olimonovarietali.it/en/region-detail/?regione=CALABRIA.

[27] Nardi M., Bonacci S., De Luca G., Maiuolo J., Oliverio M., Sindona G., Procopio A. (2014). Biomimetic synthesis and antioxidant evaluation of 3,4-DHPEA-EDA [2-(3,4-hydroxyphenyl) ethyl (3S,4E)-4-formyl-3-(2-oxoethyl)hex-4-enoate]. Food Chem..

[28] Bonacci S., Paonessa R., Costanzo P., Salerno R., Maiuolo J., Nardi M., Procopio A., Oliverio M. (2018). Peracetylation as a strategy to improve oleuropein stability and its affinity to fatty foods. Food Funct..

[29] Iriti G., Bonacci S., Lopreiato V., Frisina M., Oliverio M., Procopio A. (2023). Functional Compounds of Cold-Pressed Promegranate Seed Oil: Fatty Acids and Phytosterols Profile as Quality Biomarkers for Origin Discrimination. Foods.

[30] Herrero M., Termirzoda T.N., Segura-Carretero A., Quintares R., Plaza M., Ibañez E. (2011). New possibilities for the valorization of olive oil by-products. J. Chromatogr. A.

[31] Attia Y.M., El-Kersh D.M., Wagdy H.A., Elmazar M.M. (2018). Verbascoside: Identification, Quantification, and Potential Sensitization of Colorectal Cancer Cells to 5-FU by Targeting PI3K/AKT Pathway. Sci. Rep..

[32] Luque-Muñoz A., Tapia R., Haidour A., Justica J., Cuerva J.M. (2019). Direct determination of phenolic secoiridoids in olive oil by ultra-high performance liquid chromatography-triple quadruple mass spectrometry analysis. Sci. Rep..

[33] Kalogiouri N.P., Alygizakis N.A., Aalizadeh R., Thomaidis N.S. (2016). Olive oil authenticity studies by target and nontarget LC–QTOF-MS combined with advanced chemometric techniques. Anal. Bioanal. Chem..

[34] Kanakis P., Termentzi A., Michel T., Gikas E., Halabalaki M., Skaltsounis A.L. (2013). From olive drupes to olive oil. An HPLC-orbitrap-based qualitative and quantitative exploration of olive key metabolites. Planta Med..

[35] International Organization for Standardization (1993). Statistics, Vocabulary and Symbols.

[36] Okogeri O., Tasioula-Margari M. (2002). Changes occurring in phenolic compounds and alpha-tocopherol of virgin olive oil during storage. J. Agric. Food Chem..

[37] Esposto S., Selvaggini R., Taticchi A., Veneziani G., Sordini B., Servili M. (2020). Quality evolution of extra-virgin olive oils according to their chemical composition during 22 months of storage under dark conditions. Food Chem..

[38] Gõmez-Alonso S., Mancebo-Campos V., Desamparados Salvador M., Fregapane G. (2007). Evolution of major and minor components and oxidation indices of virgin olive oil during 21 months storage at room temperature. Food Chem..

[39] Esposto S., Taticchi A., Urbani S., Selvaggini R., Veneziani G., Di Maio I., Sordini B., Servili M. (2017). Effect of light exposure on the quality of extra virgin olive oils according to their chemical composition. Food Chem..

