# Supplementary material for: Storage Effects on Bioactive Phenols in Calabrian Monovarietal Extra Virgin Olive Oils Based on the EFSA Health Claim
Source: Foods. 2023 Oct 17;12(20):3799. doi: 10.3390/foods12203799 (PMC10606493; doi:10.3390/foods12203799)
Supplement: Supplementary file 1 [file foods-12-03799-s001.zip › foods-2661105-supplementary.pdf]

Supplementary Material

Storage Effects on Bioactive Phenols in Calabrian Monovarietal Extra Virgin Olive Oils Based on the EFSA Health Claim

Marialaura Frisina, Sonia Bonacci *, Manuela Oliverio, Monica Nardi, Thomas Patrizio Vatrano and Antonio Procopio

Department of Health Science, University Magna Græcia of Catanzaro, 88100 Catanzaro, Italy

Figure S1. Images from https://www.olimonovarietali.it/en/region-detail/?regione=CALABRIA. page 2

Figure S2. Full Scan (a) chromatogram obtained by UHPLC-ESI-HRMS for the VN sample and extracted ion chromatograms of detected EVOO phenols hydroxytyrosol (b), oleacein (c), oleocanthal (d) and oleuropein aglycon (e). page 2

Table S1. Evolution of total phenol content (mg kg^{-1}) in Nocellara del Belice (VN) and Dolce di Rossano (VDR) monovarietal EVOO samples. page 3

Table S2. Evolution of total considered biophenols during twelve months of storage expressed as mg of *EVOO phenols* per 20 g in Nocellara di Belice (VN) and Dolce di Rossano (VDR) monovarietal EVOO samples (mg/ 20 g). page 3

Figure S3. Correlation of the peak area obtained by UHPLC-ESI-HRMS and analytical standard concentration of hydroxytyrosol (a); tyrosol (b); verbascoside (c); oleacein (d); oleuropein (e); oleocanthal (f) and oleuropein aglycon (g). page 4

Table S3. Linearity validation results of the analytical method by UHPL-ESI-HRMS. page 5

Table S4. Statistical and analytical parameters: variation coefficient (CV_r); limit of repeatability (r); percentage recovery (R) and extended uncertainty (U_e) for each considered concentrations (mg g^{-1}). page 5

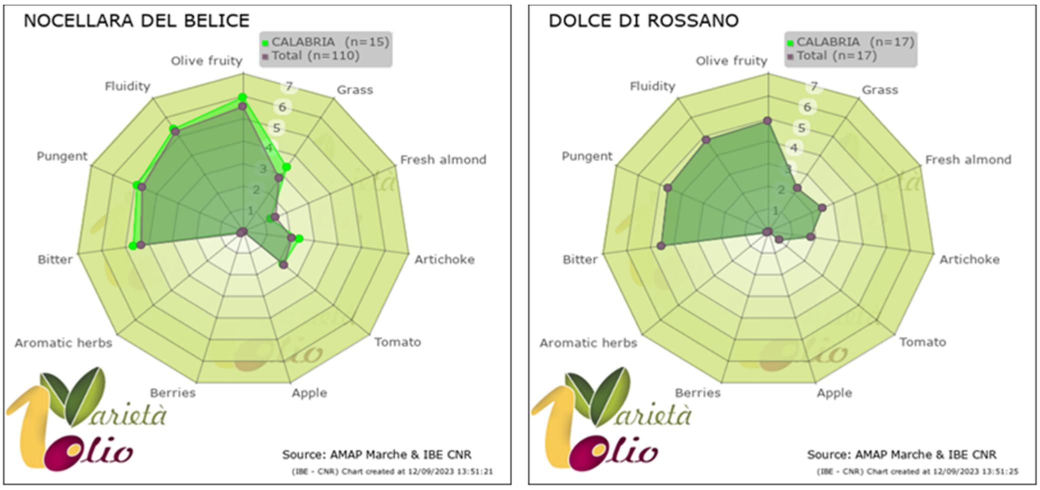

Figure S1. Images from https://www.olimonovarietali.it/en/region-detail/?regione=CALABRIA.

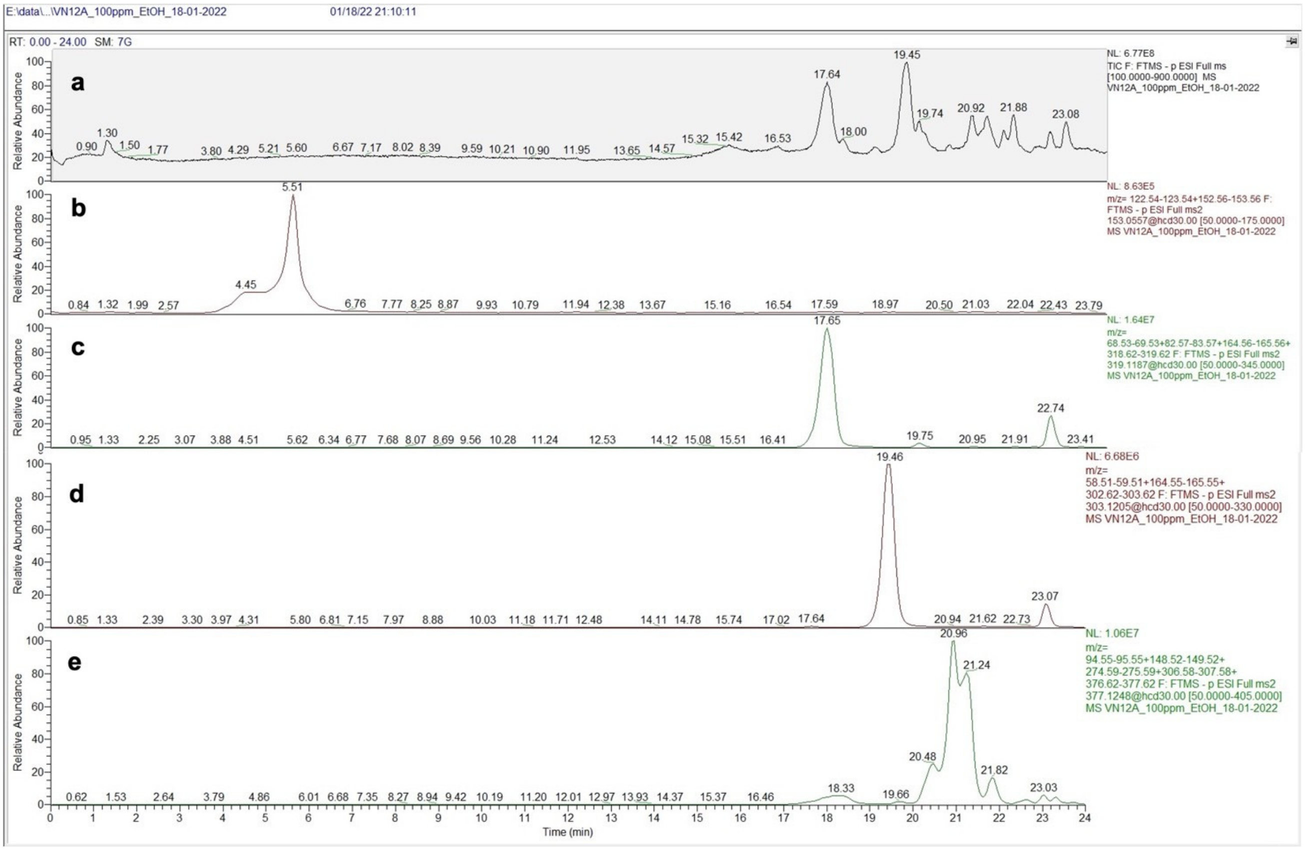

Figure S2. Full Scan (a) chromatogram obtained by UHPLC-ESI-HRMS for the VN sample and extracted ion chromatograms of detected EVOO phenols hydroxytyrosol (b), oleacein (c), oleocanthal (d) and oleuropein aglycon (e).

Table S1. Evolution of total phenol content (mg $kg^{-1} \pm$ SD) in Nocellara del Belice (VN) and Dolce di Rossano (VDR) monovarietal EVOO samples evaluated by spectrophotometric assay (Folin-Ciocalteau) during storage time (months).

	**VN*** (mg $kg^{-1} \pm$ SD)	**VDR*** (mg $kg^{-1} \pm$ SD)
**February**	472.34±9.48	566.98±18.52
**March**	552.72±15.76	486.65±16.46
**April**	475.40±15.49	501.22±22.71
**May**	486.61±10.99	486.63±10.39
**June**	532.66±12.46	429.53±8.47
**July**	523.85±13.12	554.22±15.45
**August**	506.68±20.46	569.72±14.19
**September**	510.35±11.24	340.91±23.15
**October**	529.31±12.51	513.36±15.12
**November**	511.14±19.45	624.09±21.46
**December**	509.48±10.82	535.69±10.24
**January**	499.54±17.51	555.11±14.58

*Data are expressed as means standard deviations (SD) of tree independent observations.

Table S2. Evolution of total considered EVOO phenols during twelve months of storage expressed as mg of EVOO phenols per 20 g in *Nocellara di Belice* (VN) and *Dolce di Rossano* (VDR) monovarietal EVOO samples (mg/ 20 g).

	**VN***	**VDR***
**February**	29.58±1.16	41.43±2.29
**March**	29.04±1.61	40.78±2.31
**April**	28.69±0.78	38.64±1.50
**May**	28.00±1.17	35.83±2.26
**June**	27.80±1.43	35.72±2.80
**July**	27.46±1.17	34.04±2.51
**August**	27.04±1.02	34.35±2.89
**September**	26.85±1.11	34.19±2.26
**October**	26.26±0.91	33.19±1.27
**November**	25.82±0.91	28.04±1.64
**December**	25.40±0.99	28.06±1.30
**January**	25.10±0.95	28.16±1.52

*Data are expressed as means and standard deviations (SD) of three independent observations.

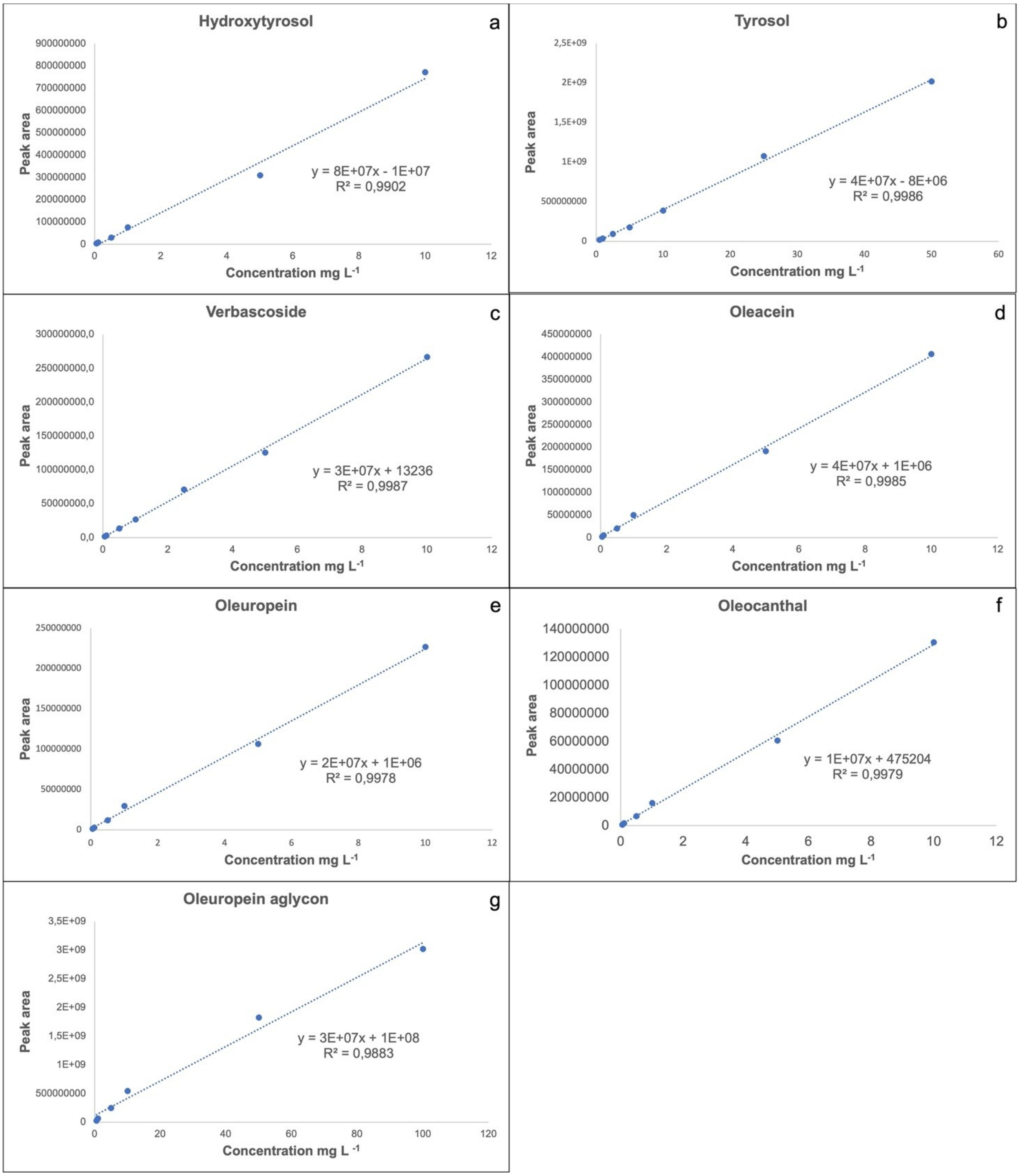

Figure S3. Correlation of the peak area obtained by UHPLC-ESI-HRMS and analytical standard concentration of hydroxytyrosol (a); tyrosol (b); verbascoside (c); oleacein (d); oleuropein (e); oleocanthal (f) and oleuropein aglycon (g).

Table S3. Linearity validation results of the analytical method by UHPL-ESI-HRMS.

Compounds	Concentration range (mg L^{-1})	Calibration curve	R^2
Hydroxytyrosol	0.05-10	Y=8E+07X-1E+07	0.9902
Tyrosol	0.5- 50	Y=4E+07X-8E+06	0.9986
Verbascoside	0.05-10	Y=3E+07X+13236	0.9987
Oleacein	0.05-10	Y=4E+07X+1E+06	0.9985
Oleuropein	0.05-10	Y=2E+07X+1E+06	0.9978
Oleocanthal	0.05-10	Y=1E+07X+475204	0.9979
Oleuropein aglycone	0.5-100	Y=3E+07X+1E+08	0.9983

Table S4. Statistical and analytical parameters: variation coefficients (CV_r and CV_R); limit of repeatability (r); percentage recovery (R) and extended uncertainty (U_e) for each considered concentrations (mg g^{-1}).

	mg g^{-1}	CV_r (%)	CV_R (%)	r (%)	R (%)	U_e (%)
	0.033	2.3	2.9	7.2	94	25
**Hydroxytyrosol**	0.037	6.1	7.2	19.4	109	20
	0.047	6.6	7.3	20.8	93	33
	0.37	2.9	3.5	9.8	97	32
**Tyrosol**	0.58	6.8	7.7	20.7	96	36
	0.75	3.3	3.9	10.5	96	31
	0.003	9.3	9.9	29.0	97	29
**Verbascoside**	0.007	8.8	9.7	27.8	98	18
	0.015	1.9	2.6	7.3	104	22
	0.09	1.7	2.4	5.5	94	33
**Oleacein**	0.13	7.5	8.0	24.0	108	31
	0.28	5.7	6.3	18.2	99	29
	0.002	9.7	10.9	32.0	89	38
**Oleuropein**	0.005	9.0	9.4	28.8	104	20
	0.013	2.1	2.8	6.2	95	23
	0.16	5.0	5.4	6.9	93	38
**Oleocanthal**	0.23	5.8	6.6	18.7	93	39
	0.53	4.4	5.2	14.2	97	26
	0.36	3.1	3.9	9.9	98	36
**Oleuropein aglycon**	0.53	6.8	7.2	21.9	95	34
	0.72	3.2	3.7	10.2	96	33
